# Is there a gradient in the association between internet addiction and health?

**DOI:** 10.1371/journal.pone.0264716

**Published:** 2022-03-03

**Authors:** Muhammad Zakir Hossin, Azharul Islam, Masum Billah, Mahjabeen Haque, Jalal Uddin

**Affiliations:** 1 Department of Global Public Health, Karolinska Institute, Stockholm, Sweden; 2 Department of General Education, Eastern University, Dhaka, Bangladesh; 3 Department of Educational and Counselling Psychology, University of Dhaka, Dhaka, Bangladesh; 4 Department of Sociology, East West University, Dhaka, Bangladesh; 5 Department of Epidemiology, University of Alabama at Birmingham, Birmingham, AL, United States of America; University of Southern Queensland, AUSTRALIA

## Abstract

**Background:**

Internet Addiction (IA) is often shown to be associated with health issues, but no study explicitly examined a possible gradient in the association between different levels of IA and health. This study aimed to examine if the levels of IA had a graded relationship with poor sleep quality, psychological distress, and self-rated health among university students in Bangladesh.

**Methods:**

In this cross-sectional study, a sample of 625 students from six universities/colleges responded to an online survey that contained measures of internet addiction test (IAT), general health questionnaire (GHQ-12), sleep quality, and self-rated health. Modified Poisson regression models were fitted to estimate the adjusted risk ratios (RR) and confidence intervals (CI) of the associations between IA and health outcomes.

**Results:**

The IA levels were associated with each of the three health outcomes in a linear fashion. Compared to the lowest IA quintile, the highest quintile remained associated with an increased risk of poor-quality sleeping (RR: 1.77; 95% CI: 1.26, 2.48), psychological distress (RR: 2.09; 95% CI: 1.55, 2.82), and worse self-rated health (RR: 1.46; 95% CI: 1.09, 1.96) after adjusting for socio-demographic covariates. There were also dose-response associations between IAT z-scores and health outcomes. The association between IAT z-scores and psychological distress was significantly stronger in males compared to females (p-value for interaction<0.05).

**Conclusions:**

The study found strong gradients between levels of addiction to internet and health outcomes, suggesting that increased health risks may exist even at lower levels of internet addiction. The findings highlight the need for departure of current research from a focus on the classic dichotomy of problematic versus not problematic internet use and a move toward recognizing the potential hierarchical effects of IA on health.

## Introduction

The uncontrolled use of the internet in the modern era has been accompanied with an addictive phenomenon known as “Internet addiction (IA)”, particularly among adolescents and young men [1–3]. IA, often interchangeably used as problematic or compulsive or pathological internet use, denotes a form of unrestricted internet use and potentially leads to adverse social, psychological, and behavioral consequences [2, 4, 5]. Although empirical investigation on this potential behavioral addiction has seen an upsurge over the last two decades [6, 7], the accumulated evidence failed to convince the American Psychiatric Association (APA) commissioned Substance Use Disorder Workgroup to consider IA as a separate behavioral addiction [8]. Instead, the Workgroup recommended Internet Gaming Disorder as a special condition for further investigation in the fifth edition of the Diagnostic and Statistical Manual of Mental Disorders (DSM-5) [9], implying the need for more research across nations.

Bangladesh has seen a dramatic growth of internet use over the last two decades, from about 0.1 million users in 2000 to over 120 million in mid-2021 [10]. Although the expansion of internet use in Bangladesh has generally been equated with economic development by policy makers, empirical evidence is now emerging to suggest that the uncontrolled use of internet among individuals, particularly among the youths, has turned into an addictive phenomenon and been associated with psychological distress and sleep disturbance [11]. However, the evidence is limited and comes mainly from studies based on purposive sampling, with small student samples restricted to one [11–13] or two [14, 15] educational institutions.

Internationally, research on IA has been growing along three overlapping perspectives: substance dependence, impulse control disorder, and relationship resource deficits [16]. In substance dependence perspective, IA is viewed as a dependence on the internet similar to the dependence on substances such as illicit drugs. Therefore, symptoms generally observed in chemical dependence are also true for IA, such as increased resource investment, unpleasant feelings, tolerance and withdrawal [17]. In the impulse control disorder perspective, IA is conceptualized as an outcome of failed control of the impulses for using the internet, as seen in pathological gambling, despite the known adverse physical, social, and psychological consequences it brings to an individual’s life [2, 5, 7, 18]. From the relationship deficits perspective, IA is considered as an artifact resulting from a deficit in the real-world relationship or lacking adequate resources to build such relationship [4, 19, 20]. Relationship deficits occur when someone prefers online interactions than in-person social interactions which are perceived to be more threatening and less controlled [4, 16, 21].

Scholars have failed to reach a consensus on the operationalization of the IA construct, posing a big challenge to meaningful comparisons across studies [16]. In the absence of a universally-accepted definition of IA, several assessment tools are being used across studies [22]. There is, however, no disagreement that the construct is multidimensional and involves at least some aspects that are common to all perspectives stated above. These shared dimensions include uncontrolled use of the internet, preoccupation, tolerance/withdrawal, mood alteration, and negative outcomes. Further, scholars have distinguished between a specific IA and a generalized or global IA [21, 23]. When someone is addicted to a specific internet feature, such as pornography or gambling, they fall under the specific IA category. A generalized or global IA refers to the addiction associated with the internet in general. The Internet Addiction Test (IAT) developed by Young [5] is widely used as a measure of global IA which encompasses all the shared dimensions of IA that the impulse control tradition proposes [22].

Studies have consistently shown a negative association between IA and sleep quality [14, 24–26]. A recent meta-analysis revealed that compared to normal internet users, problematic internet users were 2.2 times more likely to experience sleep problems [27]. Besides, the association of IA with psychiatric disorders is often documented by numerous studies, mainly using adolescents and young adult samples. Several systematic and meta-analytic studies demonstrate that IA is correlated with symptoms of attention-deficit/hyperactivity disorder (ADHD), depression, anxiety, and obsessive-compulsive and substance use disorders [28–30]. In addition to the adverse association with sleep and psychiatric disorders, IA may affect physical health because of its association with unhealthy lifestyles such as poor diets [31], physical inactivity, drinking [32], and smoking [11]. There is, however, a scarcity of evidence regarding the association of IA with general health status. One recent study explored IA in relation to the health-related quality of life, a measure that indexes physical and psychological health, and found that participants with IA scored lower in both health domains [33]. Furthermore, while the prevalence of IA is usually shown to be higher in males [1], the gender-related differences in the associations between IA and health remain underexplored [34].

Although the most popular IA measure, the Young’s IAT has been subject to poor analytic practices. Majority of the studies focused on a binary measure of problematic versus non-problematic use of the internet [35, 36]. We argue that such dichotomization may obscure significant differences and patterns in health among multiple groups of internet addicts. Different levels of IA may be associated with health outcomes hierarchically, bearing important implications for public health policy, research, and clinical practices. Only a few studies attempted to explore the association of multi-categorical IA with health [e.g., 37, 38], using Young’s classification of mild, moderate, and severe IA. To our knowledge, no study has explicitly examined the possible existence of an IA gradient in health. In the current study, we aimed to investigate if different levels of IA form any graded association with poor sleep quality, psychological distress, and general health status of the university students in Bangladesh and if the studied associations differ by gender.

## Methods

### Study population

The study employed a cross-sectional design and used data derived from a semi-structured questionnaire survey which was administered online. The target population was the students pursuing tertiary level education in Bangladesh while the study population was geographically restricted to Dhaka, the capital city of Bangladesh. Tertiary education in Bangladesh is delivered along three major streams: public university, private university, and colleges affiliated with the National University. We used a multi-stage sampling approach, with a combination of cluster sampling and random sampling, to draw a study sample from these three clusters of educational institutions. First, we selected two public universities, two private universities, and two national university affiliated colleges, using sampling frames collected from the University Grants Commission (UGC) and the National University of Bangladesh. Second, two academic departments from each of these institutions were again randomly selected. Third, we invited the students of the selected 12 departments through social media platforms to fill up the anonymous questionnaire. We aimed to include 70 students from each department, regardless of academic semesters, in the study sample. However, if the target was not achieved despite our best efforts, it was filled up by recruiting students from the other department within the same university. A total of 840 students filled out the questionnaire, of which 625 (74%) had complete data on all study variables and were subject to analyses. Data were collected during January-February 2020.

### Ethical considerations

Participation in the study was voluntary, and each participant was informed about the study purposes, its risks and benefits, and conditions of confidentiality before starting the survey, through a plain language explanatory statement. Participants then expressed their consent by clicking either “Yes” or “No”. Ethical approval (reference number DECP/O/2020/1) was obtained from the Research Ethics Committee of the Department of Educational and Counselling Psychology at the University of Dhaka.

### Measures

#### Internet addiction (IA)

We used the Bangla validated IAT scale [39] to measure IA. IAT is a 20-item scale designed to measure psychological dependence, compulsive use, withdrawal, and related problems of school, sleep, family, and time management [5]. Each item is scored on a 6-point Likert scale ranging from 0 to 5, e.g., How often do you find that you stay online longer than you intended? (0  =  Not applicable, 1  =  Rarely, 2  =  Occasionally, 3  =  Frequently, 4  =  Often, 5  =  Always). The Bangla-validation tool retained 18 items, with a possible IAT score ranging between 0 and 90. The higher the score, the greater the level of addiction. Internal consistency of the items in the study sample was found excellent (Cronbach’s alpha = .92). We used three different scales of IA as an exposure in the study. First, we divided the IAT scores into five quintiles, which resulted in an ordered categorical exposure variable. Second, we standardized the raw IAT scores to use the converted z-values as a continuous measure. Third, we also created a dichotomized PIU measure that was variedly defined as a score of 40+ or 50+ on the original IAT scale across studies [40]. Consistent with a previous study in Bangladesh [11], the threshold of PIU for the current study was set at 45 on a 90-point IAT scale.

#### Psychological distress

Psychological distress was measured by the Bangla version [41] of the 12-item General Health Questionnaire, GHQ-12 in brief [42]. The GHQ-12 was designed to screen for people’s ability to carry out normal functions and the emergence of any disturbing phenomenon, e.g., strain, depression, and losing confidence. Each item is rated on a four-point scale (less than usual, no more than usual, rather more than usual, or much more than usual). Of the different scoring procedures available for GHQ-12 [43], we followed the 0–0–1–1 scoring procedure in which the first two responses (less than usual and no more than usual) were coded as 0 and the remaining two responses (rather more than usual and much more than usual) as 1. Thus, the total score ranged from 0 to 12, with a higher score indicating higher psychological distress. The mean GHQ-12 score was previously suggested as a potential threshold to detect psychological distress [43]. As the average GHQ-12 score in the current study was 3.9, the individuals scoring > = 3.9 were defined as psychologically distressed. The internal consistency of the GHQ-12 items in the current sample was very good (Cronbach’s alpha:.84).

#### Sleep quality

For sleep quality measurement, we asked three questions, derived from DSM-5 sleep disturbance criteria, whether participants had (i) difficulty falling asleep for 30 minutes or longer (i.e., difficulty initiating sleep, DIS), (ii) woken up frequently during the night (i.e., difficulty maintaining sleep, DMS) and (iii) woken up too early in the morning and were unable to go back to sleep (e.g., early morning awakening, EMA). Each question was answered on a five-point Likert scale, from very often (1) to never (5). In this study, very often (1) and often (2) were coded as ‘poor sleep quality’ while sometimes (3), rarely (4), and never (5) were coded as ‘not poor sleep quality.’

#### Self-rated health

To measure self-rated general health status, participants were asked to rate how their overall health was on a five-point Likert scale ranging from excellent (1) to poor (5) [44]. For the current study, fair (4) and poor (5) categories were considered ‘not good health’ while excellent (1)/very good (2)/good (3) was defined as ‘good health.’

#### Sociodemographic variables

We collected data on a range of sociodemographic variables which were used as controls in the study: age (continuous), gender (female and male), place of residence during childhood (Dhaka, other cities/towns, rural area), father’s education (tertiary, secondary, and primary), parents’ marital status (married and divorced/separated/widowed), and participant’s civic status (single, partnered, married, split up/divorced). We selected these covariates based on our subject matter knowledge and the predictors of internet addiction identified in past studies [11, 45]. Thus, sociodemographic factors (e.g., age, gender, socioeconomic status, marital status) which were likely to be the common causes of both IA and health outcomes were statistically adjusted for in the regression models.

### Statistical analysis

Data analyses were performed in Stata version 15.0. The overall and gender-specific distributions of the categorical variables were presented as counts and proportions. The continuous variables were presented as means and standard deviations (SD). The detailed distributions (count, mean, SD, median and range) of the IA scores were also displayed separately for each quintile of IA. The unadjusted prevalence percentages of the three health outcomes across the five levels of IA were illustrated in graphs. The associations between IA and health outcomes were examined using the so-called modified Poisson regression models. Poisson regression has been suggested as a useful alternative to logistic regression which overestimates the relative risks when the outcome’s prevalence is common [46]. The results from the Poisson regression analysis were presented as risk ratios (RR) with 95% confidence intervals (CI). While the standard Poisson regression is usually applied to rare time-to-event outcomes to calculate the incidence rates or rate ratios, the modified Poisson regression is considered appropriate to calculate risk or prevalence ratios of outcomes with a binomial distribution. A typical assumption in Poisson regression is that the mean of the outcome equals the variance while the mean of a binary outcome exceeds the variance. Consequently, Poisson regression, when applied to a binary outcome, correctly estimates the regression coefficients but overestimates the standard errors which, however, can be easily corrected by using robust sandwich estimators of variance [46]. The RRs from the modified Poisson regression have the advantage of being more intuitive and easier to interpret than odds ratios from logistic regression [47, 48].

We fitted three regression models for each association of interest. The first model minimally adjusted for age and gender while the second model additionally adjusted for the potential sociodemographic confounders. The robustness of the confounder-adjusted associations was further assessed in a third model by mutual adjustments for all three health outcomes. The p-value for heterogeneity was obtained in post-estimation through a Wald test to assess the significance of the overall association between the IA quintiles and a specific outcome. Further, the p-value for trend was calculated to test if the IA quintiles were linearly associated with the outcomes. The binary, multi-categorical, and continuous versions of the IA measure were separately entered in the models. Our choice of the quintiles rather than the tertiles/quartiles of IA was guided by likelihood ratio tests which suggested the models with quintiles as the better fit when compared to the models with tertiles/quartiles. Moreover, before using IA as a continuous measure, we further checked possible non-linearity by introducing a quadratic term of the z-scores in a statistical model. As no departure from linearity was found, IA z-scores were additionally analyzed on a continuous scale.

The analyses were primarily carried out in the full sample combining both males and females. Besides, we presented gender-stratified results since there was evidence of effect modification by gender. The gender-stratified models, however, were minimally adjusted for age due to insufficient statistical power.

#### Missing data

Out of the study sample of 840 individuals, 25.6% had missing data on at least one of the variables used in the study (n = 215). Of all the analytic variables of interest, the exposure IA had the highest proportion of missing (19.4%) (see also **S1 Table)**. The distribution of the missing observations by the study variables is shown in **S2 Table**. It was found that the missing data did not systematically vary across any of the study variables, i.e., the missingness did not depend on the observed covariates. Given the evidence that the sample with missing data were characteristically similar to the sample with complete data, we opted for a complete case analysis under the assumption of “missing completely at random” (MCAR). The missing data is referred to as MCAR if the probability of missing depends on neither the observed variables nor the unobserved variables, although we were unable to test whether the missing data in the current study depends on any unobserved covariates.

## Results

We present the sample characteristics by gender in **Table 1**. The average age of the respondents was about 23 years (SD: 2.4). Females (54%) slightly outnumbered males (46%). Overall, about 8% of the respondents were married, 31% had an unmarried partner, 39% were single, and 22% experienced divorce or break-up. About 73% of the females, compared to 59% of the male respondents, were from Dhaka. Majority of the respondents had a father with a college degree (66%).

**Table 1:** Sample characteristics and the distribution of study variables by gender.

Characteristics	All (n = 625)	Female (n = 335)	Male (n = 290)	P-for-difference*
	% (n)	% (n)	% (n)	
**Sociodemographic covariates**				
**Age (**Mean, SD)	22.5 (2.4)	22.2 (2.3)	22.9 (2.5)	.001
**Civic status**				.001
Married	8.2 (51)	11.6 (39)	4.1 (12)	
Partnered	30.7 (292)	35.8 (120)	24.8 (72)	
Single	38.7 (242)	34.0 (114)	44.1 (128)	
Split-up/divorced	22.4 (140)	18.5 (62)	26.9 (78)	
**Place of residence in childhood**				.001
Dhaka	66.6 (416)	73.1 (245)	59.0 (171)	
Other city/town	21.1 (132)	19.4 (65)	23.1 (67)	
Rural area	12.3 (77)	7.5 (25)	17.9 (52)	
**Parents’ marital status**				.059
Married	92.3 (577)	90.5 (303)	94.5 (274)	
Divorced/separated/widowed	7.7 (48)	9.6 (32)	5.5 (16)	
**Father’s level of education**				.054
Tertiary	66.2 (414)	70.2 (235)	61.7 (179)	
Secondary/higher secondary	28.2 (176)	25.7 (86)	31.0 (90)	
Primary or less	5.6 (35)	4.2 (14)	7.2 (21)	
**Internet addiction exposures**				
**Problematic internet use**				.286
No	74.1 (463)	75.8 (254)	72.1 (209)	
Yes	25.9 (162)	24.2 (81)	27.9 (81)	
**Level of internet addiction**				
Lowest quintile	21.6 (135)	20.3 (68)	23.1 (67)	.537
2nd quintile	19.4 (121)	21.8 (73)	16.5 (48)	
3rd quintile	19.8 (124)	20.0 (67)	19.7 (57)	
4th quintile	19.5 (122)	19.1 (64)	20.0 (58)	
Highest quintile	19.7 (123)	18.8 (63)	20.7 (60)	
**Internet addiction scores** (Mean, SD)	32.4 (19.3)	32.1 (19.3)	32.7 (19.3)	.712
**Health outcomes**				
**Poor sleep quality**				.127
No	64.8 (405)	62.1 (208)	67.9 (197)	
Yes	35.2 (220)	37.9 (127)	32.1 (93)	
**Psychological distress**				.001
No	52.5 (328)	43.9 (147)	62.4 (181)	
Yes	47.5 (297)	56.1 (188)	37.6 (109)	
**Self-rated health**				.001
Good	50.1 (313)	43.6 (146)	57.6 (167)	
Fair/poor	49.9 (312)	56.4 (189)	42.4 (123)	

Note: SD = Standard deviation.

*P-values were obtained by chi-square and t-tests for categorical and continuous variables, respectively.

Over a third of the respondents reported poor quality of sleep, nearly half were found to have psychological distress (48%), and half reported their general health to be fair/poor. Gender differences in psychological distress and general health were significant, with female respondents reporting more psychological distress and poorer general health compared to their male counterparts (p < .001). The three health outcomes were weakly correlated with each other, with the highest degree of correlation found between psychological distress and self-rated health (r:.21; p < .001). The degree of correlation between sleep quality and psychological distress was .18 (p < .001). The lowest degree of correlation was found between self-rated health and sleep quality (r:.12; p < .01).

The proportion of PIUs was estimated to be approximately 26%, with no significant variation by gender. The average IA score did not differ significantly between males and females. The IA scores followed a near-normal distribution that was slightly skewed to the right, with a mean of 32.4 (SD: 19.3) and a range from 0 to 86 (**S1 Fig**). The group with the lowest level of IA scored a mean value of 9, and their score ranged between 0 and 15 on the 90-point IAT scale. On the other hand, the corresponding mean and range in the highest group was 63 and 50–86, respectively (**Table 2**).

**Table 2:** Breakdown of internet addiction scores by the five levels of internet addiction.

Level of internet addiction	Number	Mean	Std. deviation	Median	minimum	maximum
Lowest quintile	135	9.3	4.0	10	0	15
2nd quintile	121	20.0	2.7	20	16	24
3rd quintile	124	30.0	3.2	30	25	35
4th quintile	122	42.0	4.1	42	36	49
Highest quintile	123	62.7	8.9	62	50	86
**Total**	625	32.4	19.3	29	0	86

**Fig 1:**
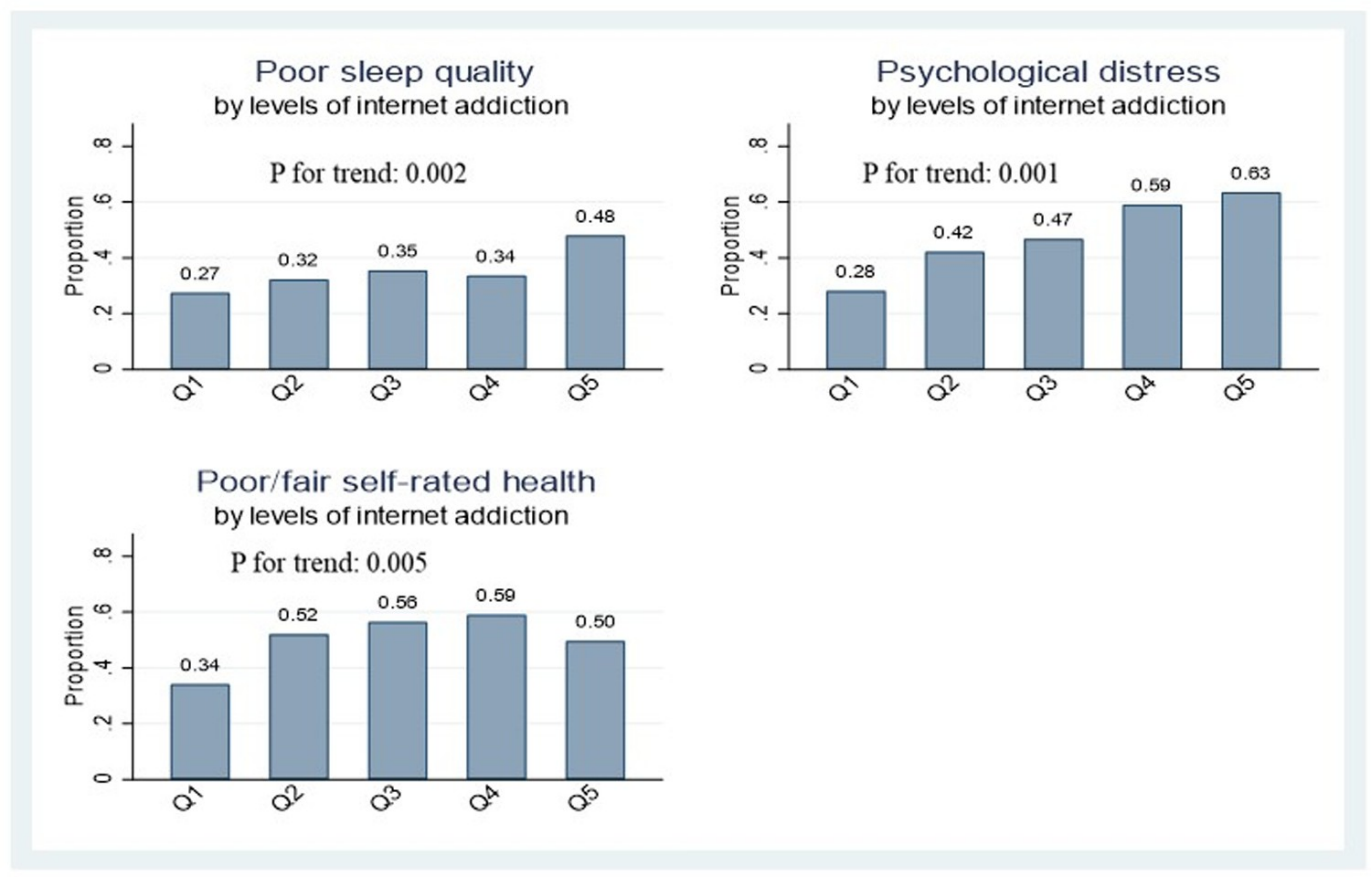
Distribution of poor sleep quality, psychological distress, and poor/fair self-rated health by levels of internet addiction (Q:quintile).

**Fig 1** presents the distribution of poor sleep quality, psychological distress, and poor/fair self-rated health by levels of IA. The prevalence of each of the three health outcomes showed a significant linear trend across the five IA levels. That is, the higher the levels of IA, the greater the prevalence of the outcome. Accordingly, about 48% of respondents in the highest IA quintile, as opposed to 27% of respondents in the lowest quintile, reported poor sleep quality. Similarly, about 63% of respondents in the highest IA quintile, in comparison to 28% of those in the lowest quintile, reported psychological distress.

The observed linear trends, however, tended to differ by gender. The trend for sleep quality was found to be statistically significant among males only. For psychological distress, the linear trend was found in both genders, but it was more strongly pronounced in females compared to males. As for self-rated health, the females showed a clear trend while the corresponding trend in the males was marginally significant (see also **Table 4**).

**Table 3** presents the estimated risk ratios (RR) and 95% CIs of the associations of IA measures with three outcomes. We found that all measures of IA–binary, quintiles, and continuous–were significantly associated with the health outcomes studied. An exception is the association between PIU and self-rated health which did not reach statistical significance. The associations followed a consistent linear pattern across all three health outcomes. Compared to the lowest IA quintile, the highest quintile was associated with an increased risk of reporting poor quality sleeping (RR:1.78; 95% CI: 1.28, 2.48), psychological distress (RR:2.21; 95% CI: 1.64, 2.98), and worse self-rated health (RR:1.43; 95% CI: 1.06, 1.92) when adjusted for age and gender. The adjustment for social covariates (i.e., father’s level of education, parental marital status, participant’s civic status and place of residence during childhood) in the statistical models made little difference to the predictive ability of IA. The studied associations largely persisted, albeit attenuated, after the mutual adjustments for the health outcomes. Although the linear trend of the association between IA levels and self-rated health disappeared in the fully adjusted model, a test for heterogeneity suggests that the IA quintiles were overall statistically significant (p < .01). There was also evidence of dose-response relationships between the IA z-scores and the health outcomes, with one unit increase in IA z-scores associated with 1.22, 1.27, and a 1.08-fold higher risk of poor sleep quality, psychological distress, and poor/fair self-rated health, respectively.

**Table 3:** Poisson regression analysis of the associations of internet addiction with poor sleep quality, psychological distress, and self-rated health (n = 625).

	**Poor sleep quality**			
**Exposure/s**	**No. of cases**	**Model 1**	**Model 2**	**Model 3**
		**RR (95% CI)**	**RR (95% CI)**	**RR (95% CI)**
**Problematic internet use**				
No	150	1.00	1.00	1.00
Yes	70	1.36 (1.09, 1.69)	1.36 (1.08, 1.70)	1.24 (0.99, 1.55)
**Level of internet addiction**				
Lowest quintile (ref)	37	1.00	1.00	1.00
2nd quintile	39	1.17 (0.80, 1.70)	1.16 (0.79, 1.70)	1.05 (0.72, 1.53)
3rd quintile	44	1.29 (0.90, 1.85)	1.27 (0.89, 1.84)	1.13 (0.79, 1.62)
4th quintile	41	1.23 (0.85, 1.79)	1.24 (0.86, 1.80)	1.03 (0.71, 1.50)
Highest quintile	59	1.78 (1.28, 2.48)	1.77 (1.26, 2.48)	1.48 (1.05, 2.08)
P for heterogeneity*		.005	.007	.066
P for trend^#^		.001	.001	.035
**Internet addiction z-scores** (per std. deviation)		1.22 (1.10, 1.34)	1.22 (1.10, 1.34)	1.16 (1.04, 1.28)
	**Psychological distress**			
**Problematic internet use**				
No	194	1.00	1.00	1.00
Yes	103	1.53 (1.29, 1.79)	1.48 (1.26, 1.73)	1.43 (1.22, 1.67)
**Level of internet addiction**				
Lowest quintile (ref)	38	1.00	1.00	1.00
2nd quintile	51	1.43 (1.02, 2.00)	1.41 (1.01, 1.97)	1.32 (0.95, 1.83)
3rd quintile	58	1.63 (1.19, 2.25)	1.60 (1.16, 2.20)	1.44 (1.05, 1.97)
4th quintile	72	2.06 (1.52, 2.79)	2.02 (1.50, 2.73)	1.84 (1.36, 2.47)
Highest quintile	78	2.21 (1.64, 2.98)	2.09 (1.55, 2.82)	1.87 (1.39, 2.51)
P for heterogeneity*		.001	.001	.001
P for trend^#^		.001	.001	.001
**Internet addiction z-scores** (per std. deviation)		1.27 (1.19, 1.37)	1.26 (1.17, 1.35)	1.22 (1.14, 1.31)
	**Poor/fair self-rated health**			
**Problematic internet use**				
No	229	1.00	1.00	1.00
Yes	83	1.04 (0.87, 1.24)	1.04 (0.87, 1.24)	0.95 (0.80, 1.13)
**Level of internet use**				
Lowest quintile (ref)	46	1.00	1.00	1.00
2nd quintile	63	1.48 (1.10, 1.97)	1.48 (1.11, 1.98)	1.42 (1.07, 1.89)
3rd quintile	70	1.64 (1.24, 2.16)	1.66 (1.26, 2.19)	1.54 (1.17, 2.03)
4th quintile	72	1.71 (1.30, 2.25)	1.73 (1.32, 2.26)	1.56 (1.19, 2.05)
Highest quintile	61	1.43 (1.06, 1.92)	1.46 (1.09, 1.96)	1.28 (0.95, 1.71)
P for heterogeneity*		.003	.002	.010
P for trend^#^		.006	.004	.084
**Internet use z-scores** (per std. deviation)		1.08 (1.00, 1.16)	1.08 (1.01, 1.17)	1.03 (0.96, 1.11)

Note: RR: Risk Ratio, CI: Confidence Interval.

Model 1: minimally adjusted for age and gender.

Model 2: Model 1 + adjusted for father’s level of education, parents’ marital status, participant’s civic status, and place of residence during childhood.

Model 3: Model 2 + mutual adjustment for sleep quality, self-rated health, and psychological distress.

*P-value for heterogeneity to test the overall association between quintiles of IA and the outcome.

^#^P-value for trend to test whether the quintiles of IA are linearly associated with the outcome.

**Fig 2:**
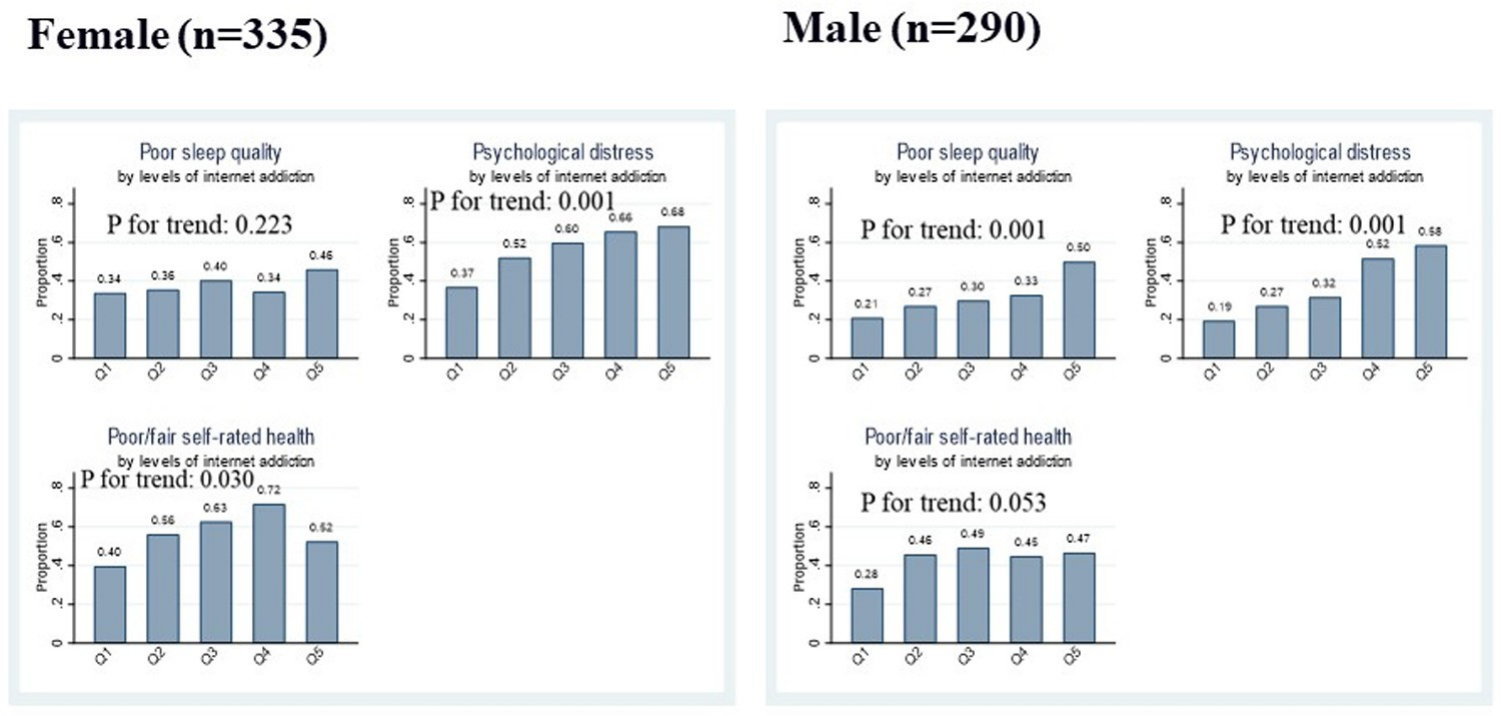
Gender-specific distributions of poor sleep quality, psychological distress, and poor/fair self-rated health by levels of internet addiction (Q:quintile).

**Table 4** presents the RR and 95% CIs of the gender-stratified associations of IA with three outcomes. The gender-stratified models were adjusted for age only. We found that the estimated associations tended to be stronger in males compared to females. For instance, the highest vs lowest quintile of IA was associated with 1.40 times higher risk of poor sleep quality in females compared to 2.83 times in males. The corresponding increased risk of psychological distress in females and males were 1.83 and 3.81, respectively. These gender differences, however, did not turn out to be statistically significant in the tests for interactions. However, the associations of PIU and IA z-scores showed significant interactions with gender regarding psychological distress. As such, one unit increase in IA z-score showed a significantly stronger association with psychological distress in males compared to females (RR: 1.20 versus 1.41). Moreover, the gender-differences in the estimated linear trends between IA quintiles and health outcomes are broadly consistent with the unadjusted trends shown in Fig 2.

**Table 4:** Gender-stratified Poisson regression analysis of the associations of internet addiction with poor sleep quality, self-rated health, and psychological distress.

	**Poor sleep quality**			
**Exposure/s**	**Female** (n = 335)		**Male** (n = 290)	
	**No. of cases**	**RR (95% CI)** ^&^	**No. of cases**	**RR (95% CI)** ^&^
**Problematic internet use**				
No	93	1.00	57	1.00
Yes	34	1.17 (0.86, 1.58)	36	1.63 (1.17, 2.26)
P for interaction^!^		.122		
**Level of internet addiction**				
Lowest quintile (ref)	23	1.00	11	1.00
2nd quintile	24	1.08 (0.69, 1.71)	18	1.56 (0.80, 3.02)
3rd quintile	26	1.10 (0.70, 1.72)	18	1.53 (0.79, 2.97)
4th quintile	25	1.08 (0.69, 1.70)	17	1.86 (0.96, 3.59)
Highest quintile	29	1.40 (0.91, 2.15)	29	2.83 (1.51, 4.95)
P for heterogeneity*		.542		.004
P for trend^#^		.176		.001
P for interaction^!^		.478		
**Internet addiction z-scores** (per std. deviation)		1.14 (1.01, 1.30)		1.32 (1.14, 1.54)
P for interaction^!^		0.115		
	**Psychological distress**			
**Problematic internet use**				
No	132	1.00	62	1.00
Yes	56	1.32 (1.09, 1.59)	47	1.93 (1.46, 2.56)
P for interaction^!^		.025		
**Level of Internet addiction**				
Lowest quintile (ref)	25	1.00	9	1.00
2nd quintile	34	1.39 (0.91, 3.62)	19	197 (0.97, 4.02)
3rd quintile	39	1.55 (1.15, 4.55)	19	1.94 (0.96, 3.94)
4th quintile	47	1.85 (1.81, 7.44)	28	3.72 (1.94, 7.12)
Highest quintile	43	1.83 (1.74, 7.44)	34	3.81 (2.01, 7.21)
P for heterogeneity*		.004		.001
P for trend^#^		.001		.001
P for interaction^!^		.153		
**Internet addiction z-scores** (per std. deviation)		1.20 (1.11, 1.30)		1.41 (1.24, 1.61)
P for interaction^#^		.040		
	**Poor/fair self-rated health**			
**Problematic internet use**				
No	143	1.00	86	1.00
Yes	46	1.01 (0.81, 1.25)	37	1.09 (0.82, 1.45)
P for interaction^!^		.607		
**Level of Internet addiction**				
Lowest quintile (ref)	27	1.00	16	1.00
2nd quintile	37	1.41 (0.98, 2.03)	28	1.62 (0.98, 2.68)
3rd quintile	43	1.58 (1.12, 2.23)	30	1.71 (1.05, 2.80)
4th quintile	49	1.79 (1.28, 2.48)	23	1.71 (1.02, 2.87)
Highest quintile	33	1.31 (0.90, 1.90)	26	1.62 (0.98, 2.68)
P for heterogeneity*		.006		.263
P for trend^#^		.035		.076
P for interaction^!^		.684		
**Internet addiction z-scores** (per std. deviation)		1.07 (0.98, 1.18)		1.09 (0.96, 1.24)
P for interaction^!^		.720		

Note: RR: Risk Ratio, CI: Confidence Interval.

^&^The estimates were minimally adjusted for age.

^!^P-value for interaction was obtained from the pooled sample by fitting an exposure*gender interaction term in the age-adjusted Poisson model.

*P-value for heterogeneity to test the overall association between quintiles of IA and the outcome.

^#^P-value for trend to test whether the quintiles of IA are linearly associated with the outcome.

## Discussion

We aimed to assess if different levels of IA had a graded relationship with poor sleep quality, psychological distress, and self-rated health among university students in Bangladesh. Results showed that IA was consistently associated with each of the three health outcomes in a linear fashion. The observed linear trend between IA and health outcomes was more strongly pronounced for psychological distress compared to poor sleep quality and self-rated health. The estimated associations and the linear trends were quite robust to the control for the participants’ sociodemographic characteristics. The association between IA and psychological distress was modified by gender, with significantly larger RR in males than in females. While the linear trend in the association of IA with psychological distress was evident in both genders, the corresponding associations with poor sleep quality and self-assessed health showed a significant linear trend in males and females, respectively.

Consistent with the previous literature [14, 27, 49], we found an increased risk of poor sleep quality associated with IA. A prospective cohort study showed that the overuse of phones (more than 4 hours per day) was associated with the incidence of sleep disturbance and mental health problems among college students during eight months of follow-up [50]. The addictive use of light-emitting gadgets (e.g., smartphones, tabs) before bedtime may extend sleep initiation time, delay the circadian clock, suppress levels of the sleep-promoting hormone melatonin, reduce the amount of rapid eye movement sleep (REMS), and decrease alertness in the morning [51, 52].

In agreement with the previous literature [11, 13, 28, 53], the current study also demonstrates that IA was strongly associated with psychological distress. Deficient self-regulation of internet use could lead to psychological distress [19]. The problematic users may end up in distress simply because of not being able to curtail internet use, even if unnecessary. To cope with this distress, the users may again engage in online activities such as unrestricted social media surfing or chatting [45]. Psychological distress could be a direct outcome of excessive internet use or a byproduct of negative consequences that IA might cause on the academic [54], behavioral [32], and social [55] lives. Moreover, the association between IA and psychological distress can be mediated by poor sleep quality because of its strong association with both IA and various mental health issues [24, 25, 56]. This is also evident in the current study that found an attenuation in the risk of psychological distress associated with IA when sleep quality was accounted for.

While past studies mostly focused on the psychological and behavioral correlates of IA, the current study adds to the literature by demonstrating that IA was strongly associated with self-rated health. Self-rated health as a proxy for an objective measure of global health status in the general population was well-validated in previous research [57]. IA may affect overall health through its associations with unhealthy lifestyle [32], poor sleep quality [27], and psychological well-being [11]. This is partly supported by the findings of the current study which showed that the statistical control for sleep quality and psychological distress weakened the association between IA and self-rated health.

Unlike most previous studies that reported higher prevalence estimates of IA in males than females [1], we did not find any gender difference in the prevalence of IA. It is plausible that the gender gap in internet use has steadily declined [58] or has started disappearing in recent times. On the other hand, the association between IA and psychological distress was found to be stronger in males. Moreover, the linear trend of the associations of IA with sleep quality and self-rated health turned out to be gender-specific. We do not know the exact mechanisms driving the observed gender differences in the associations, but these can be possibly explained by the varying patterns of internet use between males and females [58] and differential vulnerability to addictive internet use [59]. Studies have shown that males consume internet primarily for online gaming and entertainment whereas females tend to use internet primarily for interpersonal communications [58, 60–62]. Besides, females may have a greater vulnerability to online gaming disorder than males [59]. Future research should consider the purpose and duration of internet use to provide improved insights into the underlying mechanisms of the associations and their gender-specific pathways.

### Strengths and limitations

To our knowledge, this is the first study that systematically tested the possibility of a gradient between IA and health and one of the very few studies that systematically explored the potential effect modifications by gender when reporting the associations between IA and health measures. Another strength is the relatively large and multi-institute sample which provided greater statistical power and generalizability to the findings compared to previous Bangladeshi studies that recruited student samples from one [11–13] or two institutions [14, 15].

Nevertheless, our study sample may not perfectly represent the target population due to the multi-stage sampling design. Although we managed to collect a sampling frame of all the universities located in Dhaka, a sampling frame of the entire student population was difficult to obtain. We therefore opted to divide our population in clusters to collect the data. A multi-stage cluster sampling is a useful alternative to simple random sampling and is more practical and cost-efficient when the study population is vast, although representativeness might be an issue. However, our study sample is quite heterogeneous in terms of the composition of age, gender, and social class and appears to reflect the general characteristics of the larger population. A major limitation of the study is its cross-sectional design which does not allow inferring any causal direction between IA and health. It is likely that the relationships between IA and the health measures in our study are bidirectional, necessitating prospective research to disentangle the cause and effect. Moreover, all measures used in the study were self-reported and hence the possibility of bias originating from the misclassification of the exposure and outcomes cannot be ruled out. However, this is a common limitation in all surveys and not unique to our study. Another concern is the missing data which, if not missing at random, may bias the estimates. However, our exploration of the predictors of missingness based on the observed data suggests that none of the study variables are systematically correlated with missingness. If missingness does not depend on any unobserved covariates, the complete case analysis conducted in this study can be expected to produce valid estimates.

## Conclusions and implications

The study findings are suggestive of a consistent and powerful IA gradient in health, operating from top to bottom of the entire IA scale spectrum. The findings highlight the need for departure of current research and treatment approaches from a focus on the classic dichotomy of problematic versus not problematic internet use and a move toward a recognition of the potential hierarchical effects of IA on health. The standard convention has been to define PIU based on certain thresholds on some IAT scale [11, 40]. Our study challenges this tradition, given the finding that increased health risks may exist at much lower levels of addiction and not just among the so-called PIUs. Therefore, instead of relying on the classic definitions of PIU, future studies should exploit the entire IAT continuum to better identify the different risk groups (e.g., low, medium, high) and inform appropriate interventions to prevent the adverse health consequences of IA. However, to reach a broad consensus on what may constitute a safe level of internet use and for more valid insights into the observed IA gradient in health and its underlying mechanisms, we emphasize further research in other settings incorporating prospective study designs and larger study populations.

## Supporting information

S1 Table Proportion of missing observations in the study variables. (DOCX)

S2 Table Distribution of missing and complete data across the study variables. (DOCX)

S1 Fig Histogram of internet addiction test scores. (TIF)
