# Supplementary material for: Is there a gradient in the association between internet addiction and health?
Source: PLoS One. 2022 Mar 3;17(3):e0264716. doi: 10.1371/journal.pone.0264716 (PMC8893621; doi:10.1371/journal.pone.0264716)
Supplement: S1 Table — (DOCX) [file pone.0264716.s001.docx]

| **S1 Table.** Proportion of missing observations in the study variables | | |
| --- | --- | --- |
| **Study variables** | **n** | **%** |
| **Age group** |  |  |
| <20 yrs. | 73 | 8.7 |
| 20-24 yrs. | 650 | 77.4 |
| 25-30 yrs. | 117 | 13.9 |
| Missing | 0 | 0 |
| **Gender** |  |  |
| Male | 449 | 53.4 |
| Female | 387 | 46.1 |
| Missing | 4 | 0.5 |
| **Civic status** |  |  |
| Married | 70 | 8.3 |
| Partnered | 256 | 30.5 |
| Single | 329 | 39.2 |
| Split-up/divorced | 177 | 21.1 |
| Missing | 8 | 0.9 |
| **Place of residence in childhood** |  |  |
| Dhaka | 551 | 65.6 |
| Other city/town | 173 | 20.6 |
| Rural area | 106 | 12.6 |
| Missing | 10 | 1.2 |
| **Parents’ marital status** |  |  |
| Married | 770 | 91.7 |
| Unmarried | 61 | 7.3 |
| Missing | 9 | 1.1 |
| **Father’s level of education** |  |  |
| Tertiary | 545 | 64.9 |
| Secondary/higher secondary | 241 | 28.7 |
| Primary or less | 43 | 5.1 |
| Missing | 11 | 1.3 |
| **Level of internet addiction** |  |  |
| Lowest quintile | 147 | 17.5 |
| 2nd quintile | 131 | 15.6 |
| 3rd quintile | 134 | 15.9 |
| 4th quintile | 137 | 16.3 |
| Highest quintile | 128 | 15.2 |
| Missing | 163 | 19.4 |
| **Poor sleep quality** |  |  |
| No | 527 | 62.7 |
| Yes | 307 | 36.6 |
| Missing | 6 | 0.7 |
| **Psychological distress** |  |  |
| No | 415 | 49.4 |
| Yes | 363 | 43.2 |
| Missing | 62 | 7.4 |
| **Self-rated health** |  |  |
| Good health | 397 | 47.3 |
| Poor/fair health | 377 | 44.9 |
| Missing | 66 | 7.9 |
