# Supplementary material for: Is there a gradient in the association between internet addiction and health?
Source: PLoS One. 2022 Mar 3;17(3):e0264716. doi: 10.1371/journal.pone.0264716 (PMC8893621; doi:10.1371/journal.pone.0264716)
Supplement: S2 Table — (DOCX) [file pone.0264716.s002.docx]

| **S2 Table.** Distribution of missing and complete data across the study variables | | | |
| --- | --- | --- | --- |
| **Study Variables** | **Missing Data*** | **Complete Data**^#^ | **P-value** |
| **Age group** |  |  |  |
| <20 yrs. | 10.7 | 8.0 | 0.169 |
| 20-24 yrs. | 78.6 | 77.0 |  |
| 25-30 yrs. | 10.7 | 15.0 |  |
| **Gender** |  |  |  |
| Male | 46.0 | 46.4 | 0.914 |
| Female | 54.0 | 53.6 |  |
| **Civic status** |  |  |  |
| Married | 9.2 | 8.2 | 0.548 |
| Partnered | 30.9 | 30.7 |  |
| Single | 42.0 | 38.7 |  |
| Split-up/divorced | 17.9 | 22.4 |  |
| **Place of residence in childhood** |  |  |  |
| Dhaka | 65.8 | 66.6 | 0.776 |
| Other city/town | 20.0 | 21.1 |  |
| Rural area | 14.1 | 12.3 |  |
| **Parents’ marital status** |  |  |  |
| Married | 93.7 | 92.3 | 0.513 |
| Unmarried | 6.3 | 7.7 |  |
| **Father’s level of education** |  |  |  |
| Tertiary | 64.2 | 66.2 | 0.437 |
| Secondary/higher secondary | 31.9 | 28.2 |  |
| Primary or less | 3.9 | 5.6 |  |
| **Level of internet addiction** |  |  |  |
| Lowest quintile | 23.1 | 21.6 | 0.978 |
| 2nd quintile | 19.2 | 19.4 |  |
| 3rd quintile | 19.2 | 19.8 |  |
| 4th quintile | 17.3 | 20.5 |  |
| Highest quintile | 21.2 | 18.7 |  |
| **Poor sleep quality** |  |  |  |
| No | 58.4 | 64.8 | 0.095 |
| Yes | 41.6 | 35.2 |  |
| **Psychological distress** |  |  |  |
| No | 56.9 | 52.5 | 0.330 |
| Yes | 43.1 | 47.5 |  |
| **Self-rated health** |  |  |  |
| Good health | 56.4 | 50.1 | 0.167 |
| Poor/fair health | 43.6 | 49.9 |  |
| N (%) | 215 (25.6) | 625 (74.4%) |  |
| *The sample with data missing on any of the study variables.  ^#^The sample with complete data on all study variables. | | | |
