# Supplementary figures and images for: Is there a gradient in the association between internet addiction and health?
Source: PLoS One. 2022 Mar 3;17(3):e0264716. doi: 10.1371/journal.pone.0264716 (PMC8893621; doi:10.1371/journal.pone.0264716)

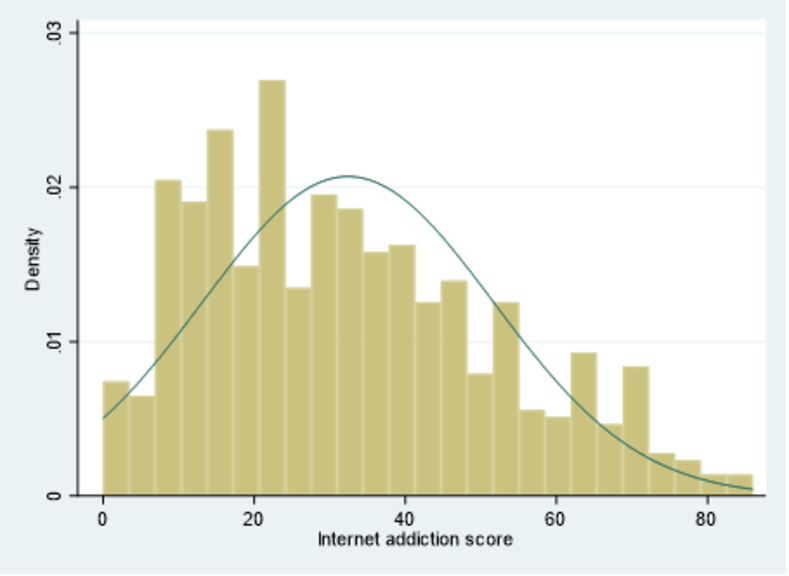

Supplement: S1 Fig — (TIF) [file pone.0264716.s003.tif]
